# Ear microbiota and middle ear disease: a longitudinal pilot study of Aboriginal children in a remote south Australian setting

**DOI:** 10.1186/s12866-022-02436-x

**Published:** 2022-01-13

**Authors:** Steven L. Taylor, Lito E. Papanicolas, Alyson Richards, Furdosa Ababor, Wan Xian Kang, Jocelyn M. Choo, Charmaine Woods, Steve L. Wesselingh, Eng H. Ooi, Patricia MacFarlane, Geraint B. Rogers

**Affiliations:** 1 Microbiome and Host Health, South Australian Health and Medical Research Institute, Adelaide, South Australia Australia; 2 College of Medicine and Public Health, Flinders University, Adelaide, South Australia Australia; 3 Department of Otolaryngology, Head & Neck Surgery, Flinders Medical Centre, Adelaide, South Australia Australia; 4 South Australian Health and Medical Research Institute, Adelaide, South Australia Australia

**Keywords:** Otitis media, Ear infection, Remote, Aboriginal, Indigenous, Microbiome

## Abstract

**Background:**

Otitis media (OM) is a major disease burden in Australian Aboriginal children, contributing to serious long-term health outcomes. We report a pilot analysis of OM in children attending an outreach ear and hearing clinic in a remote south Australian community over a two-year period. Our study focuses on longitudinal relationships between ear canal microbiota characteristics with nasopharyngeal microbiota, and clinical and treatment variables.

**Results:**

Middle ear health status were assessed in 19 children (aged 3 months to 8 years) presenting in remote western South Australia and medical interventions were recorded. Over the two-year study period, chronic suppurative OM was diagnosed at least once in 7 children (37%), acute OM with perforation in 4 children (21%), OM with effusion in 11 children (58%), while only 1 child had no ear disease. Microbiota analysis of 19 children (51 sets of left and right ear canal swabs and nasopharyngeal swabs) revealed a core group of bacterial taxa that included *Corynebacterium*, *Alloiococcus, Staphylococcus, Haemophilus, Turicella*, *Streptococcus*, and *Pseudomonas*. Within-subject microbiota similarity (between ears) was significantly greater than inter-subject similarity, regardless of differences in ear disease (*p* = 0.0006). Longitudinal analysis revealed changes in diagnosis to be associated with more pronounced changes in microbiota characteristics, irrespective of time interval. Ear microbiota characteristics differed significantly according to diagnosis (P (perm) = 0.0001). Diagnoses featuring inflammation with tympanic membrane perforation clustering separately to those in which the tympanic membrane was intact, and characterised by increased Proteobacteria, particularly *Haemophilus influenzae, Moraxella catarrhalis,* and *Oligella*. While nasopharyngeal microbiota differed significantly in composition to ear microbiota (P (perm) = 0.0001), inter-site similarity was significantly greater in subjects with perforated tympanic membranes, a relationship that was associated with the relative abundance of *H. influenzae* in ear samples (*r*_s_ = − 0.71, *p* = 0.0003). Longitudinal changes in ear microbiology reflected changes in clinical signs and treatment.

**Conclusions:**

Children attending the ear and hearing clinic in a remote Aboriginal community present with a broad spectrum of OM conditions and severities, consistent with other remote Aboriginal communities. Ear microbiota characteristics align with OM diagnosis and change with disease course. Nasopharyngeal microbiota characteristics are consistent with the contribution of acute upper respiratory infection to OM aetiology.

**Supplementary Information:**

The online version contains supplementary material available at 10.1186/s12866-022-02436-x.

## Background

Otitis media (OM) is a spectrum of pathologies that involve inflammation and/or infection of the middle ear. Characterised clinically by abnormalities of the tympanic membrane and fluid in the middle ear space, OM encompasses both acute and chronic disease, and varies in clinical presentations [1, 2]. Otitis media with effusion (OME) is common and defined as the presence of persistent fluid behind an intact eardrum, in the absence of the signs of symptoms of acute infection, while acute otitis media (AOM) involves fluid behind the eardrum with features of fever, pain, eardrum bulging, redness, or recent discharge. AOM can occur with or without perforation of the eardrum. Where persistent discharge through a perforation of the eardrum occurs for 6 weeks or more, it is defined as chronic suppurative otitis media (CSOM) [1]. All of these forms of OM are more common in childhood and are associated with increased risk of conductive hearing loss.

OM is highly prevalent in indigenous populations globally, occurring more frequently, with earlier onset, and in more severe forms, compared to non-indigenous populations [1, 3–5]. In Australia, Aboriginal children in some remote communities have prevalence rates of CSOM of 30–42% [1, 6], while non-Aboriginal Australian children have amongst the lowest rates in the world [6]. OM also has a significantly greater duration in Australian Aboriginal children, averaging 32 months compared with just 3 months in non-Aboriginal children [1]. This high burden of disease is compounded by a reliance on fly in-fly out specialist services in remote communities as well as a high turnover of resident primary health care professionals [7]. High incidence of OM in Aboriginal children contributes to higher rates of temporary and permanent hearing impairment [8], which is associated with reduced school attendance [9], poorer academic outcomes [10], and wellbeing [11], and increased likelihood of substance abuse [12] and contact with the criminal justice system [1, 13, 14].

The profound impact of OM on the long-term health and well-being of Australian Aboriginal children demands the development of more effective prevention and treatment strategies. Specific prevention, diagnosis, and treatment guidelines for OM in Australian Aboriginal and Torres Strait Islander children have been developed in recognition of the unique burden of disease and healthcare provision challenges in remote sites [15]. Of particular consideration is the microbiology of OM. Our understanding of the significance of ear microbiota characteristics, both as a contributor to aetiology and as a disease biomarker, is limited at present. Otopathogens, such as *Streptococcus pneumoniae*, *Haemophilus influenzae*, and *Moraxella catarrhalis*, are commonly identified [16, 17] and are the principal targets of medical intervention through antibiotic therapy. Notably, rates of otopathogen colonisation in Indigenous children with OM are particularly high and CSOM appears to be associated with a different microbiological profile compared to other OM conditions [18]. Prospective cohort studies in Australian Indigenous children have also reported nasopharyngeal carriage of these principal otopathogens to be a risk factor for OM in some studies [19, 20], but not all [17]. Moreover, medical interventions including liberal antibiotic prescription and vaccination programs have had limited effectiveness in indigenous populations [21–23].

Achieving a better understanding of the relationships between ear and nasopharyngeal microbiology and OM clinical course would inform efforts to reduce incidence and improve outcomes in Aboriginal children living in remote settings. There is an increasing appreciation that otopathogens exist in a wider context of other bacteria that can influence risk and outcome of OM, both positively or negatively [17, 18, 24]. To this end, our aim was to explore OM presentation and microbiology in children attending an outreach clinic in a remote community in South Australia, including disease characteristics between and within children over a two-year period, and to relate these to characteristics of the ear canal and nasopharyngeal microbiota.

## Methods

### Ethical approval and consent to participate

Ethics approval for this study was obtained from the Southern Adelaide Clinical Human Research Ethics Committee (249.15) and the Aboriginal Health Research Ethics Committee (04–15-615) and all methods were carried out in accordance with relevant guidelines and regulations (declaration of Helsinki). Children in the Yalata community in South Australia were examined by a visiting specialist ear nose and throat surgeon at the Tullawon Health Clinic as part of a pre-existing outreach programme. With the assistance of nursing staff and Aboriginal Health Workers (AHW) from the Aboriginal Health Service, parents and carers of children in the community were informed of the visits, and parents/caregivers were encouraged to bring their children to the clinic for assessment. With the assistance of AHW, informed consent to participate in the study was obtained from parents/guardians once the research project had been explained to them in either their local dialect (Pitjantjatjara) or English. During specialist visits to the community, children underwent a physical examination and age-appropriate hearing screening.

### Study setting and population overview

The Yalata region is on Aboriginal owned land on the edge of the Nullarbor Plains, approximately 200 km from the nearest regional centre and 800 km from metropolitan Adelaide. The community is composed of several family groups. The population in the community fluctuates as people move between surrounding communities for work, education, medical, ceremonial, and familial reasons. At the 2016 national census, there were approximately 200 Aboriginal people living in the community, one-quarter of whom where under the age of 14. There is a Community Controlled Health Service, staffed by full time Aboriginal Health Works, Remote Area Nurses and with regular visiting general practitioner, specialist, and allied health services.

**Fig. 1:**
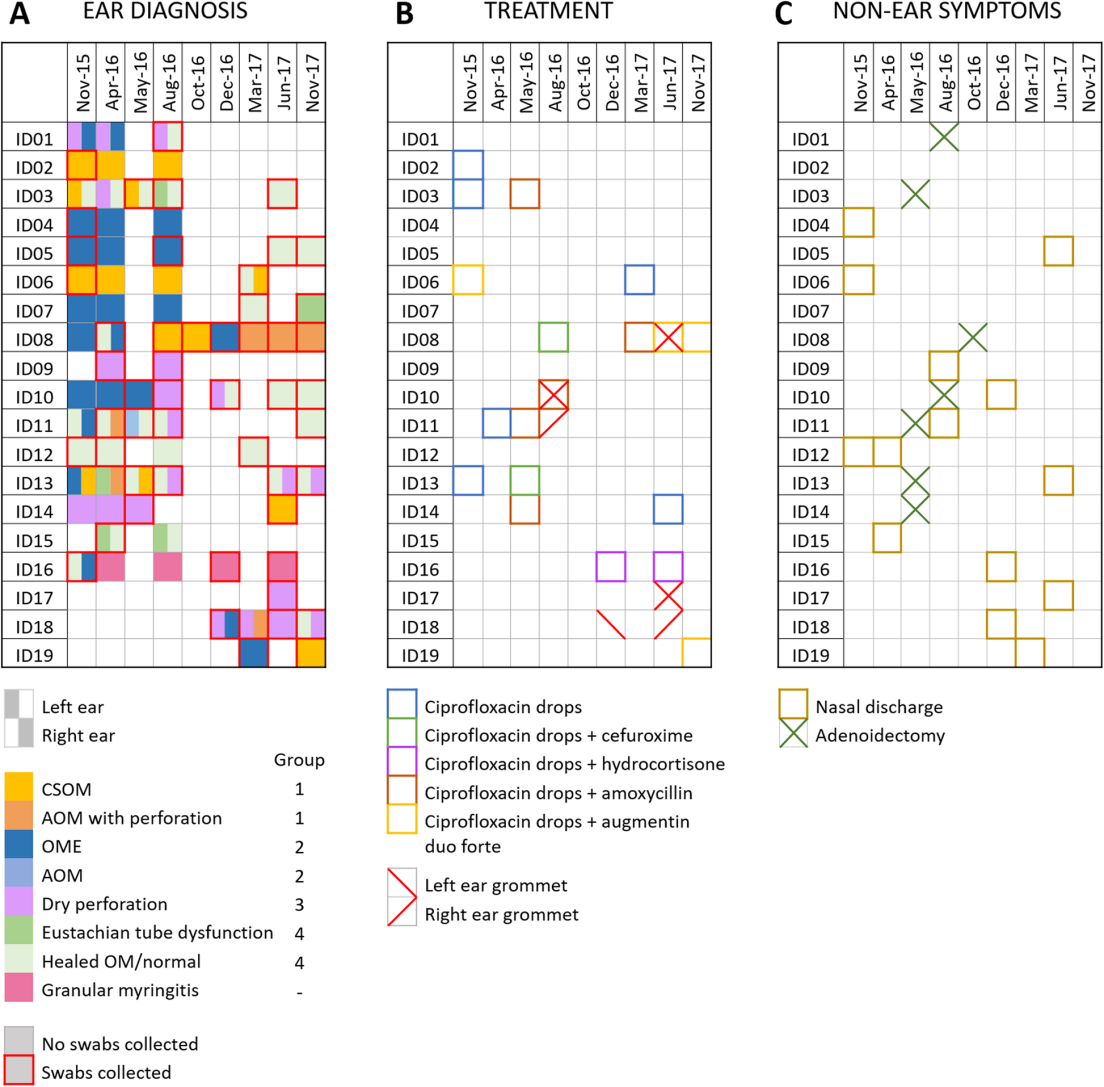
Longitudinal sample collection, otitis media (OM), ear treatment and symptom overview. **A** Diagnosis of otitis media during outreach clinic. Chronic suppurative otitis media (CSOM), acute otitis media (AOM), otitis media with effusion (OME). Group corresponds to OM disease and tympanic membrane perforation. Granular myringitis samples excluded from all OM disease group analysis. **B** Treatments prescribed or performed during outreach. C) Signs of nasal discharge and adenoidectomy recorded during visit

Participants were 19 children aged 3 months to 7 years at time of enrolment, with longitudinal samples collected from 15 participants. Samples were collected over a 2-year period (Nov 2015-Nov 2017). Participant demographics and sample collection information are described in Table 1 and Fig. 1. Vaccination records were available for 14/19 participants, with all 14 having vaccinations that were up to date with their age group.

**Table 1:** Participant demographics and clinical information during study period

Variable	Number
Number of participants	19
Age at first visit, median years (min, max)	3.20 (0.23, 8.75)
Female, n (%)	10 (53%)
Aboriginal or Torres Strait Islander, n (%)	19 (100%)
Vaccination status, n (%)	
Up to date	14 (74%)
Unknown	5 (26%)
Number of participants with > 1 timepoint, n (%)	14 (74%)
Number of visits with swabs collected, median (min, max)	2 (1, 7)
Days between first and last swab (in those with > 1 timepoint), median (min, max)	485 (150, 730)
Participants with OM during study period, n (%)	
CSOM	7 (37%)
AOMwP	4 (21%)
AOM	1 (5%)
OME	11 (58%)
Participants with other ear symptoms during study period, n (%)	
Dry perforation	
Eustachian tube dysfunction	4 (22%)
Granular myringitis	1 (5%)
Participants with no OM or ear symptoms during study period, n (%)	1 (5%)
Participants receiving pharmaceutical treatment during study, n (%)	
Ciprofloxacin drops	10 (53%)
Oral antibiotics	9 (47%)
Participants receiving surgical treatment during study, n (%)	
Grommet insertion	5 (26%)
Adenoidectomy	7 (37%)

Abbreviations: *AOM* acute otitis media, *AOMwP* acute otitis media with perforation, *CSOM* chronic suppurative otitis media, *OM* otitis media, *OME* otitis media with effusion

### Sample collection

Anonymised bilateral ear and nasopharyngeal swabs were taken from those children enrolled in the study, who were present in the community at the time of each outreach visits. Ear canal swab collection consisted of Copan flocked swabs (FLOQSwabs® 553C) inserted into the ear canal as close to the tympanic membrane as the child would permit. Nasopharyngeal swab collection consisted of the same flocked swabs inserted into the nasal cavity along the floor of the nose and directed posteriorly towards the nasopharynx as far as tolerated by the child. Effort was made to minimise contact with external skin to avoid contaminating skin flora. All swabs were stored without storage media (dry) and placed directly into the clinic freezer for the duration of the visit. Swabs were transported on ice in an insulated ice chest for up to 4 hours before being stored at − 80 °C until processed. Culture-based analysis of the swabs was not performed as swabs were frozen soon after collection to preserve microbial DNA.

### Otitis media and hearing assessment and management

All children who presented to the clinic underwent a medical assessment and a screening hearing assessment, regardless of whether they were enrolled as a participant in the study. OM was diagnosed by the visiting otolaryngologist specialist. Clinical and audiological data were correlated and if medical intervention was required then children were commenced on appropriate oral and/or topical therapy for OM. Targeted medical therapy followed the therapeutic guidelines that were current at the time of the child being seen. Escalation of therapy occurred if children had been prescribed standard medical treatment prior to specialist review by staff in the health clinic, and if there was ongoing evidence of chronic ear discharge.

For the purpose of assessing the association between ear microbiology and ear disease, ear condition was categorised into four discrete diagnosis groups. Group 1 included OM with inflammation with tympanic membrane perforation (e.g. CSOM, AOM with perforation). Group 2 included of inflammation without perforation (e.g. OME, AOM). Group 3 included no inflammation with tympanic membrane perforation (e.g. dry perforation). Group 4 included no inflammation and no perforation (e.g. normal or healed tympanic membrane).

### DNA extraction and 16S amplicon sequencing

Swab DNA was extracted as described previously [25] using an extraction method involving heat, mechanical, enzymatic and chemical lysis steps, followed by column-based DNA purification (see online supplement).

The V4 hypervariable region of the bacterial 16S rRNA gene was amplified from sputum DNA using the primers: 515F (5′-GTGCCAGCMGCCGCGGTAA-3′) and 806R (5′-GGACTACHVGGGTWTCTAAT’) with Illumina adapter overhang sequences as previously described [26]. Amplicons were generated, cleaned, and indexed, and paired end sequencing was performed according to the Illumina MiSeq 16S Metagenomic Sequencing Library Preparation protocol. Sequencing data are deposited in the European Nucleotide Archive database (PRJEB42298).

### Bioinformatic processing

Sequence output was demultiplexed using QIIME2 [27] (release 2019.4). The DADA2 plugin was used to trim, de-replicate, merge, and remove chimeric sequences, as well as identify and correct sequencing errors [28]. Representative sequences were aligned to the SILVA database (v132) at 80% using vsearch and unassigned sequences were filtered out. Remaining unique amplicon sequence variants (ASVs) were classified using the QIIME2 sklearn algorithm to the SILVA database trimmed to the V4 region of the 16S rRNA gene at 97% sequence similarity. The resulting taxa (ASVs that aligned to SILVA to the genus level or higher) were then examined. Taxa that were Mitochondria, Chloroplast, Cyanobacteria, or Archaea were filtered out, as were taxa present at < 1% relative abundance in at least 1 sample. Following taxa filtering, it was noted that some of the bacterial taxa identified are potential contaminants [29]. However, these taxa are also plausible constituents of the samples and as such these taxa were denoted by an asterisk (*) where presented in our findings. Samples with fewer reads than the rarefaction plot asymptote (600 reads) were removed, which included 3 ear swabs and 3 nasopharyngeal swabs.

### Quantitative PCR

Enumeration of bacteria *Moraxella catarrhalis* and *Haemophilus influenzae,* and fungi *Candida albicans* and *Aspergillus fumigatus* were performed using quantitative PCR (qPCR) as described previously [30–33] and provided in detail in the online supplement.

### Statistical analysis and visualisation

Genus-level relative abundance data were square-root transformed and Bray-Curtis similarity scores calculated using the vegan R package (version 2.5–7). Between group differences in Bray-Curtis scores were analysed using permutational multivariate analysis of variance (PERMANOVA). Principle co-ordinate analysis (PCoA) and non-metric multi-dimensional scaling (nMDS) plots were used to visualise Bray-Curtis distances, calculated using vegan and visualised using ggplot2 (version 3.3.3). Linear discriminant analysis (LDA) Effect Size (LEfSe) was used to assess relative abundance differences between groups, using one-against-all multi-class analysis, and cut-offs of LDA ≥3 and *p* < 0.05. Kruskal–Wallis test with Dunn’s post-hoc analysis used to validate LEfSe findings, as well as compare species level qPCR results and age between groups.

## Results

### Cohort overview and otitis media prevalence

Fifty-one sets of swab samples (left ear canal, right ear canal, and nasopharynx) were collected from 19 children presenting to Tullawon Health Clinic in remote Western South Australia over a two-year period. Multiple sets of samples were collected longitudinally from 19 children with the age range at the time of first swab collection was between 3 months and 8.7 years (median [IQR] = 3.2 [1.7–4.9], Table 1).

The middle ear and hearing health status for each child was assessed at every visit during which samples were collected (Fig. 1A). From a total of 102 ear diagnoses (left and right ear during 51 examinations), signs of OM with symptomatic tympanic membrane perforation were identified in 22 cases (15 CSOM and 7 AOM with perforation or AOM with grommet), asymptomatic tympanic membrane perforations were diagnosed in 20 (12 dry perforation, 8 dry grommet), signs of OM without perforation in 16 (1 AOM, 15 OME), and no signs of current disease or perforation in 36 (15 healed OM, 21 normal ear). Granular myringitis was diagnosed in 4 cases (2 visits of bilateral disease in 1 participant) and eustachian tube dysfunction in 4 cases from 3 participants. Rates of unilateral and bilateral OM presentation were similar (42 and 47%, respectively).

Treatment was primarily ciprofloxacin drops alone (*n* = 8), or ciprofloxacin drops with oral antibiotics (*n* = 7), and surgical insertion of grommets (*n* = 9). An overview of all interventions is provided in Fig. 1B and non-ear symptoms in Fig. 1C.

### Disease heterogeneity within ear samples longitudinally

Within-participant changes in diagnosis are presented as a network plot (Supplementary Fig. 1). In the majority of cases, diagnoses were consistent between visits, with 74 of 114 (65%) of consecutive examinations resulting in the same diagnosis, compared to 40/114 instances where diagnosis changed (Supplementary Fig. 1). Of the instances where diagnosis changed, OME recovered to normal four times, dry perforation recovered to healed four times, while there were three instances where OME progressed to CSOM, and three instances where dry perforation progressed to CSOM. Overall, however, no one transition disease phenotype was predominant.

### Ear microbiota characteristics

**Fig. 2:**
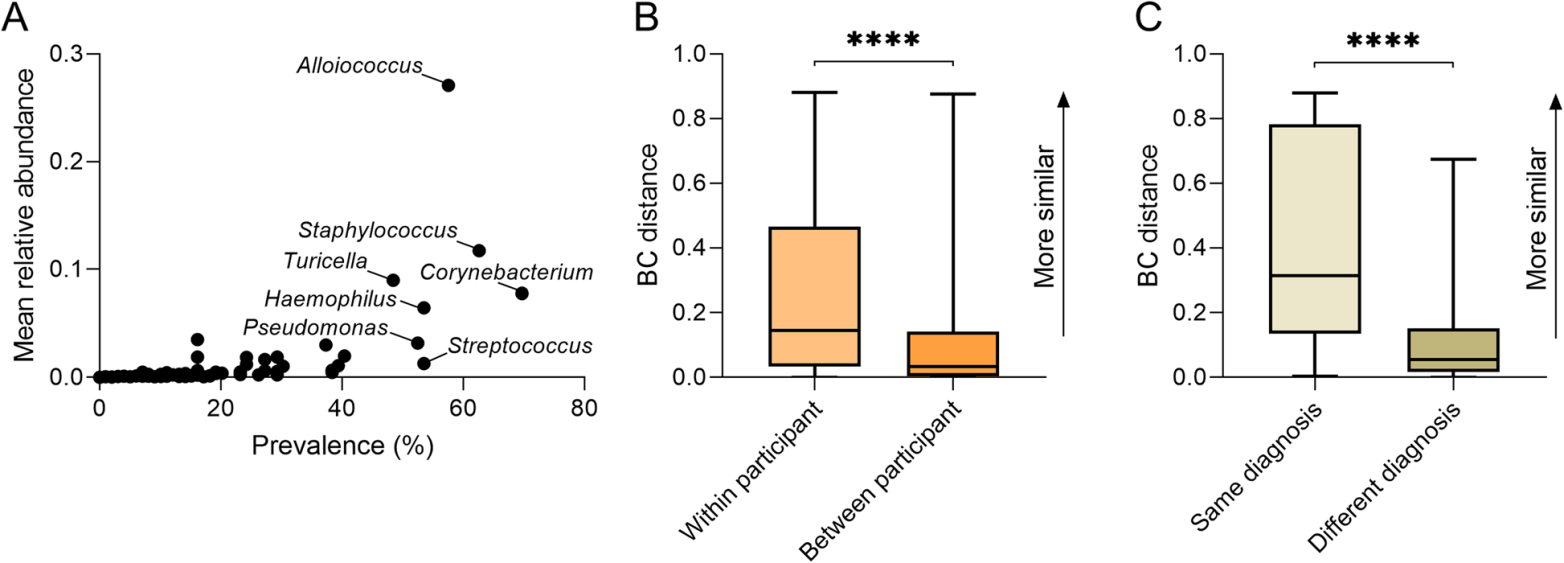
**A** Mean relative abundance and detection prevalence (%) of taxa in ear swabs. Labelled taxa indicate presence in at least 50% of ear swab samples. **B** Bray-Curtis similarity (BC distance) of ear microbiota comparing swabs collected within individuals to those collected between individuals, **C**) Longitudinal ear microbiota similarity for ears that had the same diagnosis between visits vs ears that had different diagnoses between visits

Analysis of the ear swab microbiota identified 134 bacterial taxa at ≥1% relative abundance in at least one sample. Of these, 20% were Actinobacteria, 14% Bacteroidetes, 35% Firmicutes, 25% Proteobacteria, and 6% other bacterial phyla. A median of 40 taxa were identified within any individual sample (IQR: 30–53), representing approximately 30% of all detected taxa. While this finding is consistent with considerable variation in microbiota composition between samples, several taxa were commonly detected. *Corynebacterium* was identified most frequently (70% of ear swabs at a mean relative abundance of 7.8%), while *Alloiococcus,* was found at the highest mean relative abundance (present in 58% of ear swabs at a mean relative abundance of 27%). *Staphylococcus, Haemophilus, Turicella*, *Streptococcus*, and *Pseudomonas* were all detected in at least 50% of samples and had mean relative abundances of 11, 6.5, 9.0, 1.3, and 3.2%, respectively (Fig. 2A). Taxa bar plots of all ear and nose samples are presented in Supplementary Fig. 2. Analysis of fungi *Candida albicans* and *Aspergillus fumigatus* was also performed. However, only *A. fumigatus* was detected and only in one ear swab.

### Variation in microbiota composition within and between individuals

Similarity in ear microbiota were calculated for all samples using a Bray-Curtis similarity matrix (BC distance), where a distance score between 0 and 1 indicates similarity (most similar = 1, least similar = 0). Based on the first sample collected from each participant, the microbiota of a subject’s ear was more similar to their other ear than to swabs from other participants (median BC distance score within participant = 0.22 (IQR: 0.52–0.07) vs between participant = 0.04 (IQR: 0.14–0); *p* = 0.0006). This finding remained true when all time points were assessed (median BC distance score within participant = 0.14 (IQR: 0.46–0.03) vs between participant = 0.03 (IQR: 0.14–0); *p* < 0.0001, Fig. 2B). When stratified according to clinical diagnosis (e.g. CSOM, AOMwP, AOM, OME, dry perf, eustachian tube dysfunction or normal), microbiota similarity was greatest between the left and the right ear for an individual when the diagnosis was the same. However, even when the diagnoses were different, microbiota similarity was still significantly higher within an individual compared to between individuals, and was non-significantly higher than between individuals, even where individuals had the same diagnosis (Supplementary Fig. 3A).

Longitudinal analysis of ear swab microbiota similarity was then performed. Where ear swabs were compared between two timepoints, they were more similar if the diagnosis remained the same, as compared to instances where diagnosis changed (Fig. 2C). This finding was unaffected by the time interval between examinations, with a weak correlation between time between samples and BC distance (Supplementary Fig. 3B). Taken together, these findings indicate that both participant heterogeneity and disease diagnosis contribute to observed ear microbiota.

### Ear microbiota, OM symptoms, and tympanic membrane integrity

**Fig. 3:**
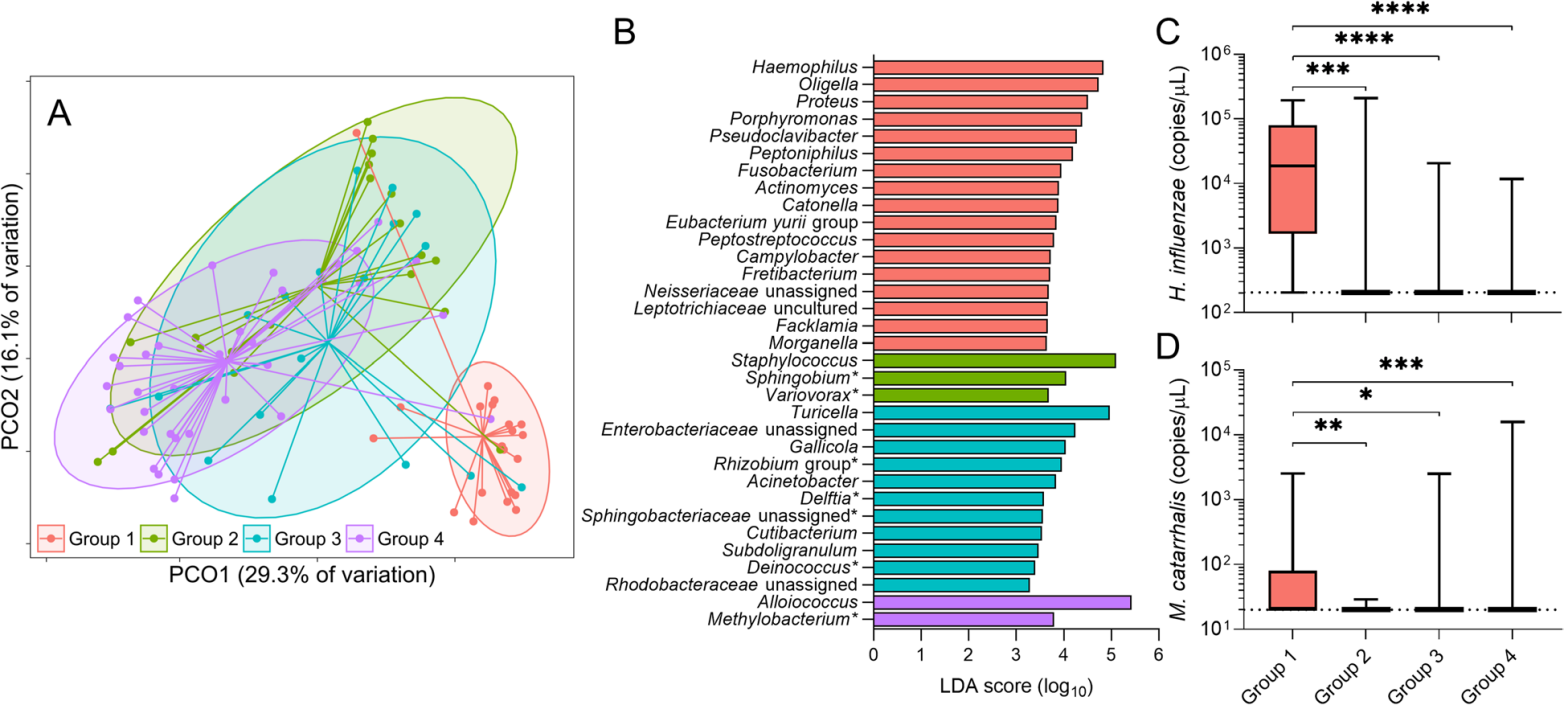
**A** Principle Coordinate Analysis (PCoA) plot of ear microbiota of all samples, grouped by ear diagnoses. Group 1 (red): Ear disease with tympanic membrane perforation, Group 2 (green): Ear disease with intact tympanic membranes, Group 3 (blue): No ear disease with tympanic membrane perforation, Group 4 (purple): No disease and intact tympanic membrane. **B** Bacterial taxa that differed significantly between ear diagnosis groups. Performed by linear discriminant analysis effect size (LEfSe). **C** Absolute load of *Haemophilus influenzae* between groups. **D** Absolute load of *Moraxella catarrhalis* between groups. * indicates taxa that are commonly associated with reagent contaminants

Ear microbiota characteristics were assessed relative to four discrete diagnosis groups: 1) inflammation with tympanic membrane perforation (*n* = 21); 2) inflammation without perforation (*n* = 20); 3) no inflammation with tympanic membrane perforation (*n* = 18); 4) no inflammation and no perforation (*n* = 36). Microbiota composition differed significantly between Groups (PERMANOVA: *r*^2^ = 21.2%, P (perm) = 0.0001). Of note, Group 1 (inflammation with tympanic membrane perforation) showed the greatest difference to the other Groups (Supplementary Table 1) and was found to cluster separately (Fig. 3A). It should be noted that the age and sex profiles of the four groups differed (Supplementary Fig. 3C and 3D). Those in diagnosis Group 1 and Group 2 were significantly younger than Group 3 and Group 4, while females had a higher prevalence of having perforated ear disease (Group 1) over the study period.

The microbiota composition of the ear swabs is shown in Supplementary Fig. 4, grouped according to the four diagnosis categories. LEfSe analysis identified 33 taxa as contributing significantly to inter-group differences. For example, the genus *Haemophilus* was particularly prevalent in Group 1, while *Staphylococcus*, *Turicella*, and *Alloiococcus* were predominant in Groups 2, 3 and 4, respectively (Fig. 3B). These findings were confirmed by Kruskal–Wallis test with Dunn’s post-hoc analysis (Supplementary Fig. 5). Exploratory species level analysis for *Staphylococcus* spp. identified that the relative abundance of *Staphylococcus aureus* was significantly higher in Group 2 and Group 4 compared to Group 1 (Supplementary Fig. 6). Of note, *Oligella*, a Gram-negative, aerobic Proteobacteria, which is not commonly associated with human respiratory or ear infections [34], was a substantial contributor to Group 1 microbiota characteristics. Targeted qPCR-based assessment of *H. influenzae* and *M. catarrhalis* carriage confirmed absolute abundance of these species to be higher in Group 1 compared to other diagnostics groups (Fig. 3C-D).

### Nasopharyngeal microbiota characteristics

**Fig. 4:**
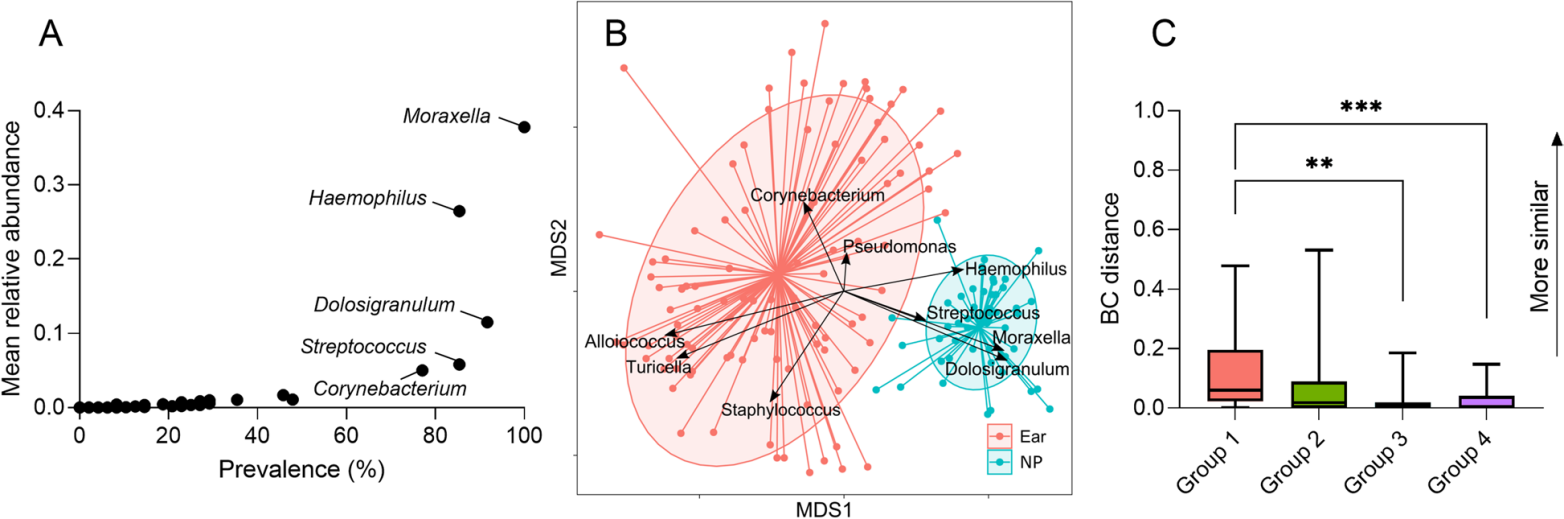
**A** Mean relative abundance and detection prevalence (%) of bacteria in nasopharyngeal (NP) swabs. **B** Non-metric multi-dimensional scaling (NMDS) biplot of ear and nasopharyngeal microbiota. **C** Bray-Curtis similarity (BC distance) between nasopharyngeal microbiota and ear microbiota, separated by ear disease groups. Group 1: Ear disease with tympanic membrane perforation, Group 2: Ear disease with intact tympanic membranes, Group 3: No ear disease with tympanic membrane perforation, Group 4: No disease and intact tympanic membrane

Microbiota profiles generated from nasopharyngeal swabs were sparser and more consistent between subjects than ear sample profiles. Eighty-eight taxa were detected across all nasopharyngeal swabs, of which 53 were detected at ≥1% relative abundance in at least one swab. Compared to ear swabs, more taxa were common across samples, with a median of 37 taxa per swab (IQR: 29–48). These taxa represented Firmicutes (31%), Proteobacteria (31%), Actinobacteria (17%), Bacteroidetes (16%), and other phyla (5%). *Moraxella* was detected in all nasopharyngeal samples and displayed the highest mean relative abundance at 38% (Fig. 4A, Supplementary Fig. 2). *Haemophilus, Dolosigranulum, Streptococcus*, and *Corynebacterium* were all detected in at least 50% of samples with mean relative abundances of 26, 12, 5.8, and 5.0%, respectively (Fig. 4A, Supplementary Fig. 2).

Nasopharyngeal microbiota differed significantly in composition to ear microbiota (PERMANOVA: *r*^2^ = 24.5%, P (perm) = 0.0001). This difference was driven primarily by higher relative abundance of *Alloiococcus*, *Turicella*, *Corynebacterium*, and *Pseudomonas* in ear swabs and higher relative abundance of *Moraxella*, *Dolosigranulum*, *Streptococcus*, and *Haemophilus* in the nasopharyngeal swabs (Fig. 4B).

Nasopharyngeal microbiota showed little similarity to ear microbiota for individual participants (median BC distance = 0.015 (IQR: 0.06–0). However, this relationship differed between diagnosis groups, with nasopharyngeal microbiota more similar to the ear microbiota in Group 1 (inflammation with tympanic membrane perforation) compared to Group 3 (no inflammation with tympanic membrane perforation) or Group 4 (no inflammation, no perforation) (Fig. 4C). The abundance of *Haemophilus* in Group 1 ear swabs was the likely principal contributor to this, with reciprocal abundance typical in nasopharyngeal swabs. To explore this further, *Haemophilus* relative abundance in ear and nose swabs were correlated to the BC distance between these swabs for Group 1 samples. Relative abundance of *Haemophilus* in the 21 ear swabs with inflammation with tympanic membrane perforation was positively correlated with BC distance between ear and nasopharyngeal sample (*r*_s_ = 0.71, *p* = 0.0003), indicating that high *Haemophilus* in the ears was associated with a greater similarity between ear and nasopharyngeal microbiota.

### Relationships between disease, treatment, and ear microbiota over time

Microbiota characteristics from a single individual (ID08), collected on seven successive occasions during a 19-month period (aged 7 months to 2 years 2 months), were related to disease characteristics and treatment. During this period, an initial presentation of unilateral OME progressed to bilateral CSOM, reverted to OME, and then developed into AOM with perforation (Fig. 1). In response, a step-up therapy approach was employed. No treatment was prescribed for initial OME, ciprofloxacin drops plus oral cefuroxime was prescribed at first presentation with CSOM (the diagnosis of which was based upon reported duration of discharge) (Fig. 1), and ciprofloxacin drops with oral amoxicillin once AOM with perforation was diagnosed. As symptoms continued, grommets were inserted, and oral amoxicillin continued. At the last study visit, oral co-amoxiclav (amoxicillin/clavulanic acid) and topical ciprofloxacin were prescribed.

**Fig. 5:**
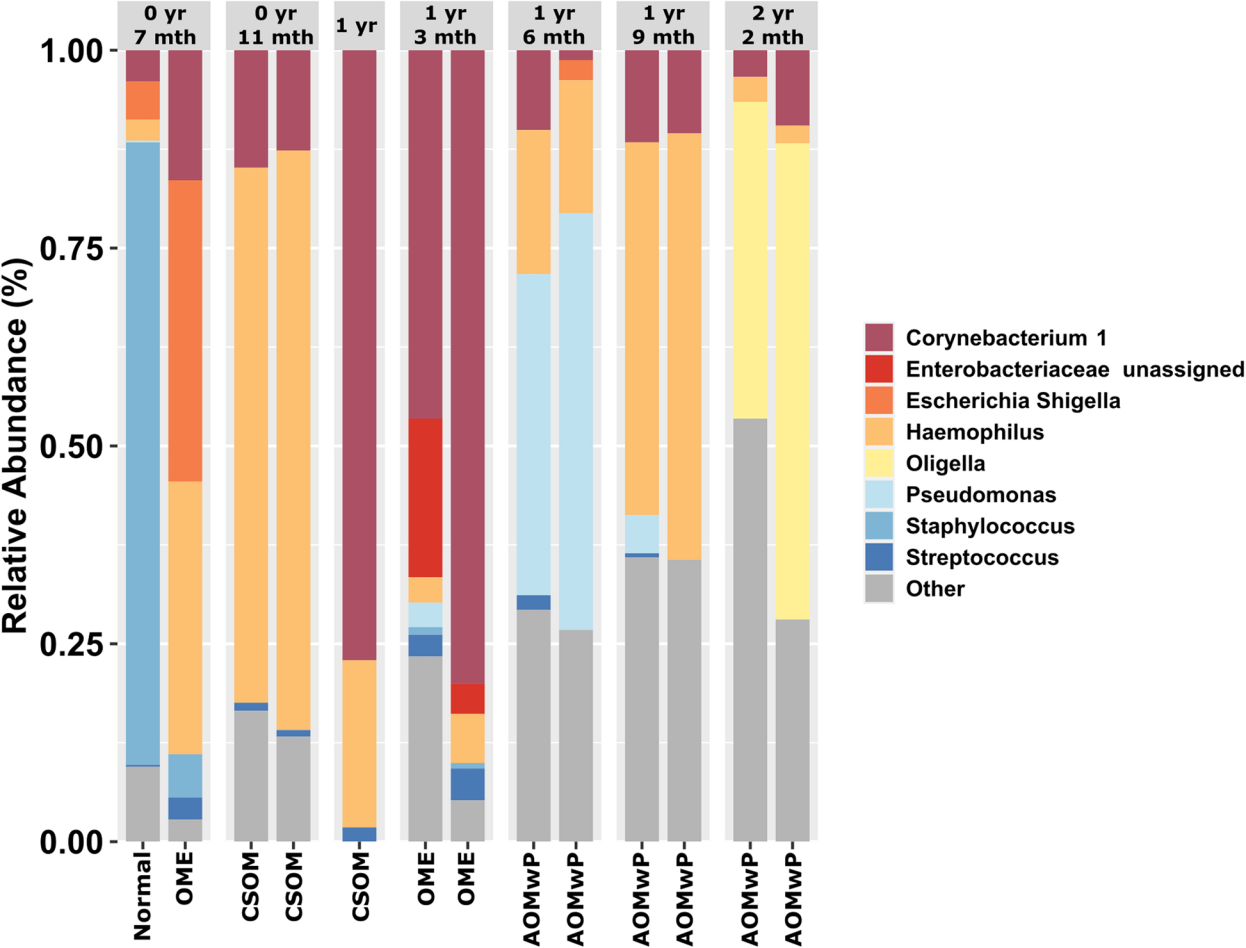
Taxa bar plot of longitudinal ear swab microbiota for participant ID08 showing changes in predominant taxa. Chronic suppurative otitis media (CSOM), otitis media with effusion (OME), acute otitis media with perforation (AOMwP)

Changes in clinical signs and treatment were reflected in ear canal microbiology (Fig. 5). Initial unilateral OME was associated with a high prevalence of *Staphylococcus* (78%). Throughout interventions for bilateral CSOM the predominant taxa changed from bilateral *Haemophilus* (68–73% relative abundance) to *Corynebacterium* (47–80% relative abundance) once the tympanic membranes had healed and OME developed, to *Pseudomonas* (41–52%) when AOM with perforation was observed. At the time of procedure, when grommets were inserted, the relative abundance of *Pseudomonas* had declined (0–5%), while *Haemophilus* relative abundance increased reciprocally (47–54%). Finally, at the consult 5 months after grommet insertion, *Oligella* was prevalent in both ear swabs (40–60% relative abundance).

## Discussion

We report an exploration of the incidence and clinical and microbiological characteristics of OM in children presenting at a South Australian outreach clinic over a two-year period. The aim of this study was to provide a basis to re-examine OM prevention and treatment strategies for this community and remote Aboriginal communities more widely.

Study participants exhibited ear disease that aligned with other studies of Aboriginal children living in remote communities, over a third of children in this study exhibiting CSOM over the study period [5]. OME was also seen in over half of these children. The types of ear disease exhibited by study participants varied with age and comparable rates of unilateral and bilateral OM presentations are also consistent with national data [35].

Recognition of the potential for a wider spectrum of bacteria to contribute to OM pathology, as well as for the wider microbial communities to positively and negatively influence the behaviour of pathogens [17, 24], has resulted in a growing application of microbiota-wide analysis to OM samples [18]. Most commonly, these take the form of 16S rRNA gene amplicon sequencing, which, when applied in an appropriate manner [24, 36, 37], can provide detailed compositional data relating to all bacteria present. The composition of the ear microbiota reported here are broadly consistent with previous studies. We identified *Alloiococcus*, *Staphylococcus*, and *Turicella* as prevalent, in keeping with studies of middle ear fluid of children living in metropolitan regions with recurrent AOM [24], as well as Aboriginal children in Central Australia [36]. Whether *Alloiococcus* and *Turicella* are otopathogenic or part of the normal aural “flora” remains contentious [24]. We found that *Alloiococcus* was more prevalent in the ear canals of children without current OM, while *Turicella* was more prevalent in those with either no OM or a dry perforation. *Staphylococcus aureus*, on the other hand, was significantly over-represented in ear canals of those with OME and was significantly higher than those with either CSOM or AOM with perforation. Further research is required to determine whether *S. aureus* is indicative or contributory to OME.

In keeping with previous studies in Aboriginal children in remote Australia [17], and globally [38, 39], we found *H. influenzae*, and to a lesser extent, *M. catarrhalis*, to be associated with chronic and perforated OM. We also found *Oligella* to be significantly more abundant in those with ear disease with perforation, detected in 16% of all ear swabs and with a relative abundance up to 60%. *Oligella* consists of 2 Gram-negative, aerobic species associated with urosepsis, *Oligella urethralis* and *Oligella ureolytica*. However, *Oligella* is rarely associated with OM [34] although it has been reported at high abundance in middle ear swabs from indigenous Filipinos with chronic otitis media [40]. The potential for *Oligella* to be an important contributor to OM in Aboriginal children clearly requires further investigation. Indeed, it is notable that ciprofloxacin-resistant clinical isolates of *Oligella* have been reported in a number of clinical contexts [41–43].

Based on longitudinal analysis, we reported that ear microbiota composition varies greatly within individuals. In participant ID08, for example, the predominant organism varied between *Corynebacterium*, *Pseudomonas*, *Haemophilus*, and *Oligella*, within a 11-month window. These shifts in which organisms were predominant also co-occurred with changes in diagnosis and treatment. Whether changes in microbiology preceded changes in disease, or vice versa, remains unclear. However, these findings do suggest that high rates of OM in Aboriginal communities cannot be explained by a single un-recognised otopathogen.

It has been suggested that acute upper respiratory tract infections (URTI) may represent an important contributory factor in OM development, directly through infection of the middle ear, by promoting the build-up of fluid in the middle ear, or by promoting the proliferation of opportunistic pathogens within the upper respiratory tract [44, 45]. Rates of URTI are significantly higher in Aboriginal children [46] and might therefore contribute to high OM incidence. There was little similarity between nasopharyngeal swab and ear swab microbiota composition, in keeping with previous studies [24, 36]. However, we did find that the ear canal microbiota was more similar to the nasopharyngeal microbiota in those children in whom the tympanic membrane had been ruptured (Group 1). This increase in similarity was driven largely by a high relative abundance of the otopathogen, *H. influenzae*, which trended strongly towards increased relative and absolute abundance in nasopharyngeal samples of those with respiratory symptoms. Disruption of commensal microbiology and local immune homeostasis by acute respiratory viral infection can increase the relative abundance of opportunist bacterial pathogens and the likelihood of bacterial infection in the upper respiratory tract and adjoining regions such as the Eustachian tube and middle ear cleft [44]. Therefore, whether high rates of URTI in Aboriginal children, including those caused by respiratory viral infection, are causally associated with high rates of bacterial infection in the middle ear and lower respiratory tract, should be considered.

It is notable that the bacterial species detected in our analysis, including those present in the ear canal in children with perforated tympanic membranes, sit within the spectrum of activity of standard antibiotic therapies for OM [15]. Where OM is recurrent, the surgical insertion of a grommet into the tympanic membrane can be used to prevent the build-up of fluid in the middle ear (a measure that was performed in just over a quarter of the study participants during the period of investigation). Therefore, rather than standard therapies being inappropriate for this population, high OM incidence, duration, and severity are likely to reflect a combination of high exposure to environmental risk factors, poorly understood treatment adherence, and limited access to specialist otolaryngology services [47–49].

Our study had limitations that should be considered. Microbiological analysis was based on molecular analysis from swab samples collected from the ear canal, with culture-based analysis not performed. While OM diagnosis and antibiotic prescription are based largely on otoscopy and assessment of signs and symptoms, ear canal swabs can be used as a basis of diagnostic microbiology, particularly where treatment response has been poor, there is exudate in the ear canal, or where there is otitis externa (inflammation of the passage of the outer ear). Ear canal swabs clearly do not sample material from the middle ear in those with intact tympanic membranes. Sampling middle ear fluid through myringotomy would require sedation or a general anaesthetic, which is not standard care unless examination requires surgical intervention. Particularly in remote communities such as Yalata, the required resources for such procedures is not available for therapeutic or research practices. Despite the sample site being ear canals, studies comparing the ear canal microbiota to the middle ear fluid of children with AOM identified surprisingly similar composition between the two sites, although *Haemophilus* was under-represented in the ear canal [24]. Being a safe, well-tolerated, and readily employed approach, ear canal samples, when combined with a history of duration of discharge, do have potential applicability as a representative sample of the middle ear microbiota in the context of perforation, or even as a prognostic indicator. In addition to bacterial and fungal pathogens, respiratory viruses can contribute substantially to OM development and progression [16], however viruses were not investigated in this study. Assessment of antibiotic susceptibility was not part of our study. However, the ability to detect the presence of antimicrobial resistance with a specific child or within a community would allow for tailoring of antimicrobial choice, and such data would inform our understanding of treatment efficacy. Finally, all analysis was performed without the adjustment for confounding factors such as age or sex owing to the exploratory nature of this study. Further, larger studies relating the ear microbiome to OM are required to assess the contribution of age and sex to these associations.

## Conclusions

OM has a considerable and long-lasting impact on the health, education and the future prospects of Australian Aboriginal children, particularly for those living in remote communities. Addressing this disease burden is complex and challenging, requiring consideration of a wide range of potential contributors, including genetic, environmental, socioeconomic and demographic factors, access to healthcare, and microbial ecology. Our use of a microbiome-focused molecular strategy to describe the heterogeneity and variance of ear canal microbiota and its associations with disease and treatment, highlight the potential utility of such an approach as part of the ongoing wider effort to better understand, prevent, and manage OM in Aboriginal children.

## Supplementary Information

**Additional file 1.**

## Data Availability

The dataset generated and analysed during the current study is available in the European Nucleotide Archive database (PRJEB42298; available at www.ebi.ac.uk/ena/browser/view/PRJEB42298) and additional data is available from the corresponding author on request.

## Abbreviations

AHW: Aboriginal Health Workers
AOM: Acute otitis media
AOMwP: Acute otitis media with perforation
ASV: Amplicon sequence variant
BC Distance: Bray-Curtis similarity distance
OM: Otitis media
CSOM: Chronic suppurative otitis media
LEfSe: Linear discriminant analysis effect size
nMDS: Non-metric multi-dimensional scaling
OME: Otitis media with effusion
PERMANOVA: Permutational multivariate analysis of variance
PCoA: Principle co-ordinate analysis
qPCR: Quantitative PCR
rRNA: Ribosomal ribonucleic acid
URTI: Acute upper respiratory tract infections
