# Supplementary material for: Ear microbiota and middle ear disease: a longitudinal pilot study of Aboriginal children in a remote south Australian setting
Source: BMC Microbiol. 2022 Jan 13;22:24. doi: 10.1186/s12866-022-02436-x (PMC8756658; doi:10.1186/s12866-022-02436-x)
Supplement: Supplementary file 1 — Additional file 1. [file 12866_2022_2436_MOESM1_ESM.docx]

**DNA extraction**

Bacteria were removed from swabs by vortexing in 400 µL of 10 mM Tris and 1 mM EDTA for 30 s. Heat lysis was performed by incubating at 95 °C for 1 min and cooled on ice for 1 min. Mechanical lysis was performed by bead beating using silica/zirconium beads at 1:1 ratio of 0.1 and 1.0 µm using a FastPrep-24 instrument at 6.5 m/s for 1 min. Enzymatic lysis was performed by incubation for 1 h at 37 °C in 2.9 mg/ml lysozyme and 0.14 mg/ml lysostaphin (Sigma-Aldrich, St. Louis, MO, USA) and 30 min at 56 °C in 1.2 mg/mL Proteinase K (ThermoFisher Scientific, Victoria, Australia), 2.5% w/v 5 M NaCl, and 1.5% w/v sodium dodecyl sulphate (Sigma-Aldrich, MO, USA). Chemical lysis and organic matter separation was performed by phenol:chloroform:isoamyl alcohol (25:24:1; saline buffered at pH 8.0; Sigma-Aldrich, MO, USA), added at 1:1, and vortexing for 30 s. The aqueous-organic layers were separated by centrifugation at 13,000 x g for 10 min and 400 μL of the aqueous layer was transferred to a new microfuge tube. DNA was recovered using an EZ-10 Spin column in accordance with manufacturer’s instructions (Bio Basic, Inc., Ontario, Canada), following precipitation by the addition of 10 M ammonium acetate and 99% ethanol (Sigma Aldrich, MO, USA) in a 1:10 and 1:1 ratio with sample volume, respectively. DNA was eluted in 50 μL UltraPure DNase/RNase-free distilled water (Gibco, ThermoFisher Scientific, Victoria, Australia) and stored at -80 °C prior to analysis.

***Quantitative PCR***

*Moraxella catarrhalis* was assessed using SYBR Green assay with 1 µL of DNA extract, 0.2 µM of forward (5'-GTGAGTGCCGCTTTTACAACC) and reverse (5'-TGTATCGCCTGCCAAGACAA) primers, 17.5 µL of PowerUp SYBR (ThermoFisher Scientific, Vic, Australia), and the appropriate volume of water to a 35 μL total reaction volume. Cycling conditions for were: 50 °C for 2 min, 95 °C for 10 min, followed by 40 cycles of 95 °C for 15 s and 60 °C for 1 min.

*Haemophilus influenzae* was assessed using TaqMan assay with 1 µL of DNA extract, 0.25 µM of forward (5'-ATTAAATGTTGCATCAACGC) and reverse (5'-GACTTTTGCCCACGCAC), 0.2 µM of probe (FAM-ACGRTTTTACCATAGTTGCACTTTCTC-BHQ1), 17.5 µL of 2X KAPA Probe Fast qPCR Master Mix (KAPA Biosystems Inc., Wilmington, USA) and the appropriate volume of water to a 35 μL total reaction volume. Cycling conditions for were: 50 °C for 2 min, 95 °C for 10 min, followed by 40 cycles of 95 °C for 10 s and 63 °C for 30 s.

*Candida albicans* was assessed using TaqMan assay with 1 µL of DNA extract, 0.2 µM of forward (5'- CGGGTGGGAAATTCGGT) and reverse (5'- CRRTGATCGGTATCGGGT), 0.1 µM of probe (FAM- CAGCTTGTAGTAAAGAATTACTCAC -BHQ1), 17.5 µL of 2X KAPA Probe Fast qPCR Master Mix (KAPA Biosystems Inc., Wilmington, USA) and the appropriate volume of water to a 35 μL total reaction volume. Cycling conditions for were: 50 °C for 2 min, 95 °C for 10 min, followed by 40 cycles of 95 °C for 10 s and 58 °C for 60 s.

*Aspergillus fumigatus* was assessed using SYBR Green assay with 1 µL of DNA extract, 0.2 µM of forward (5'-GAAAGGTCAGGTGTTCGAGTCA) and reverse (5'-TTTGGTTGCGGGTTTAGGGATT) primers, 17.5 µL of PowerUp SYBR (ThermoFisher Scientific, Vic, Australia), and the appropriate volume of water to a 35 μL total reaction volume. Cycling conditions for were: 50 °C for 2 min, 95 °C for 10 min, followed by 40 cycles of 95 °C for 15 s and 60 °C for 1 min.

All quantitative real-time PCRs were performed on three technical replicates, at 10 μL reaction volume per replicate, on a QuantStudio 6 and 7 Flex Real-Time PCR system (Applied Biosystems, Carlsbad, USA). Standard curves were generated for each qPCR reaction based on serial dilutions of ATCC strains of known concentrations of *M. catarrhalis* and *H. influenzae*.

| **Supplementary Table 1:** Pairwise permutational multivariate analysis of variance | | | | |
| --- | --- | --- | --- | --- |
| **Pair 1** | **Pair 2** | **t** | **P(perm)** | **Unique perms** |
| Group 1 | Group 2 | 3.04 | 0.0001 | 9946 |
| Group 1 | Group 3 | 2.61 | 0.0001 | 9922 |
| Group 1 | Group 4 | 4.64 | 0.0001 | 9933 |
| Group 2 | Group 3 | 1.51 | 0.0246 | 9929 |
| Group 2 | Group 4 | 2.08 | 0.0015 | 9937 |
| Group 3 | Group 4 | 2.08 | 0.0013 | 9936 |


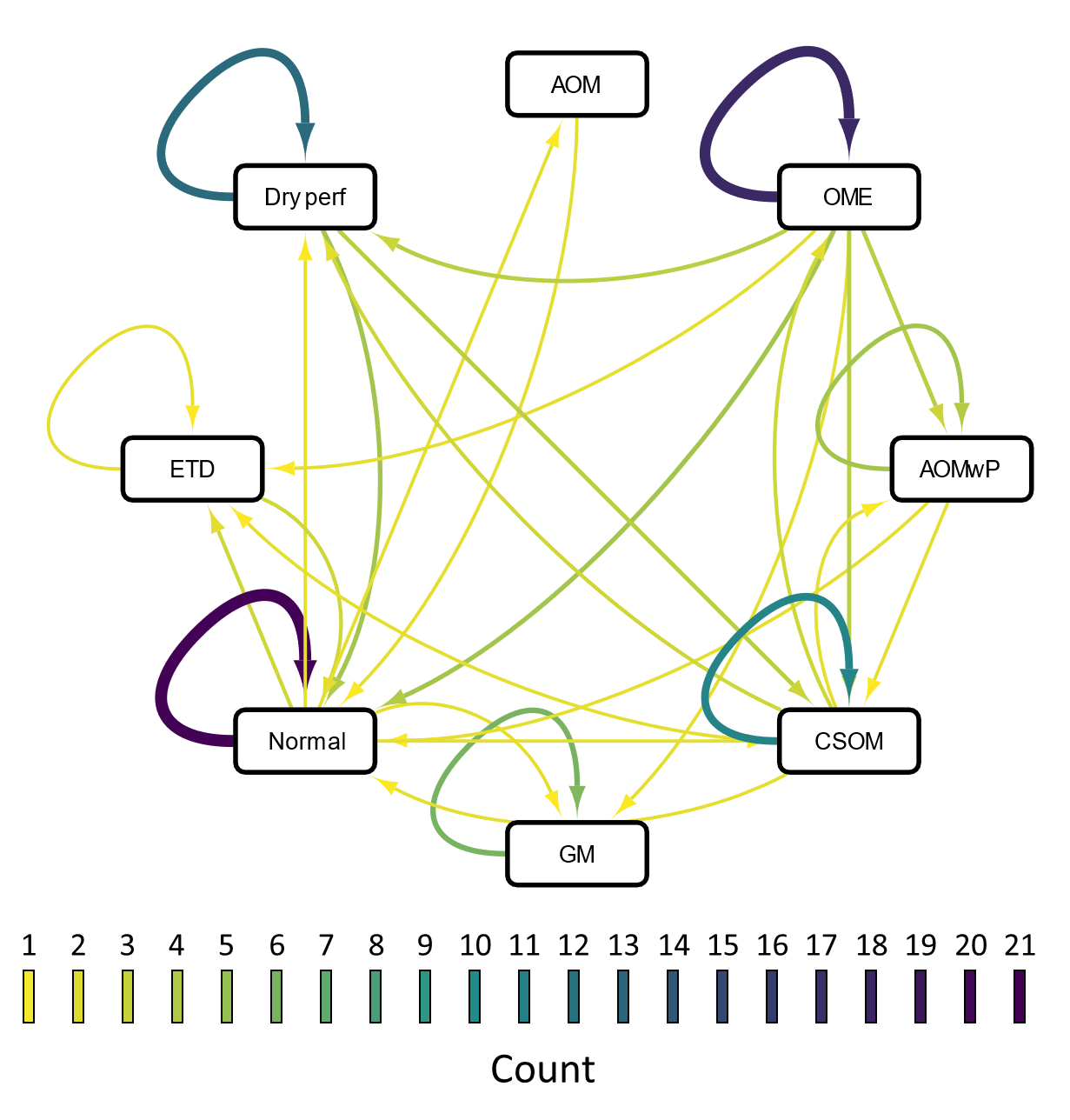


**Supplementary Figure 1:** Network diagram of the incidence of repeat ear diagnoses or change in ear diagnoses. Colours of lines represent the frequency a diagnosis changed or remained the same and the direction of the arrow indicates the direction of the change. Acute otitis media (AOM), otitis media with effusion (OME), Acute otitis media with perforation (AOMwP), chronic suppurative otitis media (CSOM), granular myringitis (GM), no ear disease (Normal), eustachian tube dysfunction (ETD), dry perforation (Dry perf).


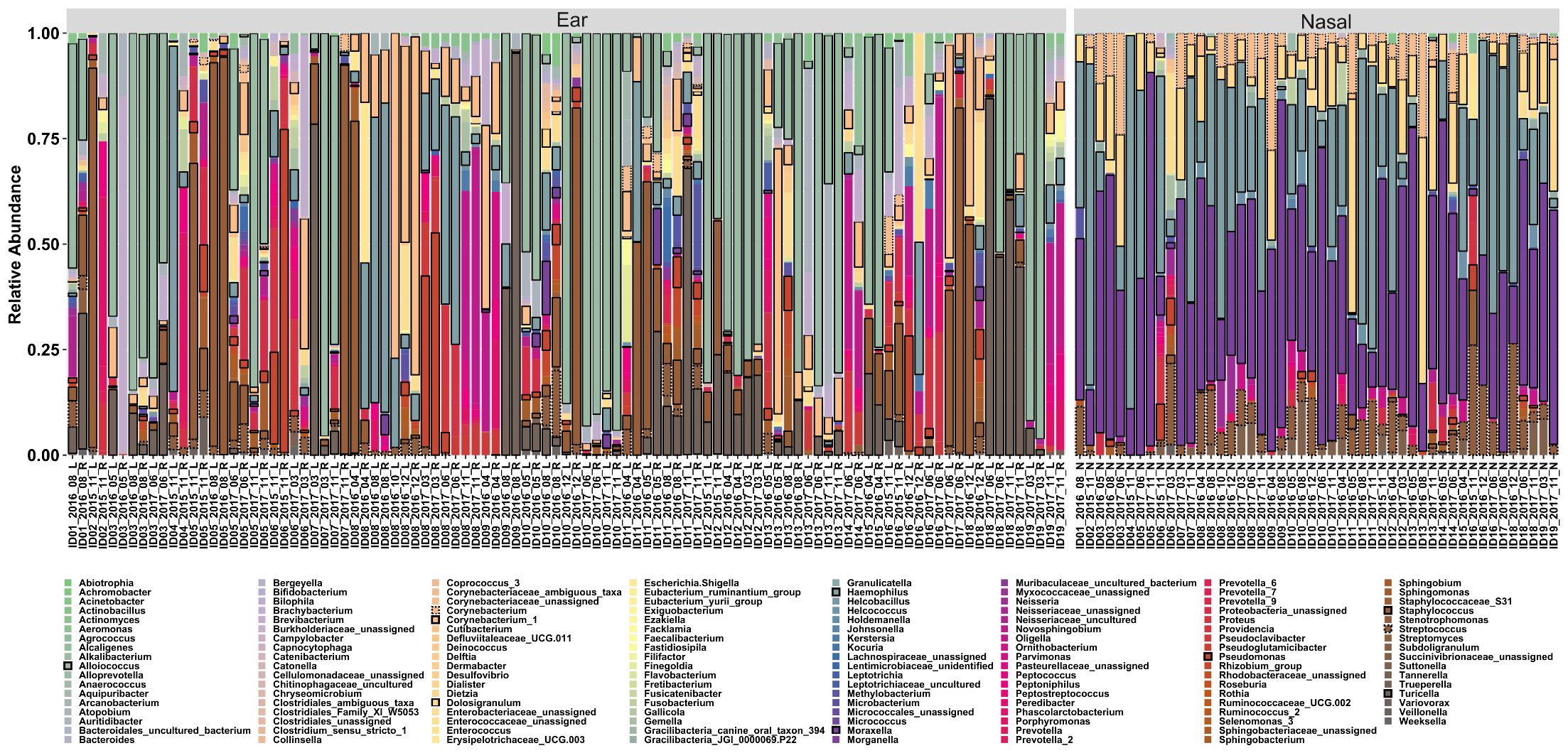


**Supplementary Figure 2:** Taxa bar plot of ear and nasopharyngeal swabs. Taxa highlighted by bold are those present in either >50% of ear swabs or >70% of nasopharyngeal swabs.


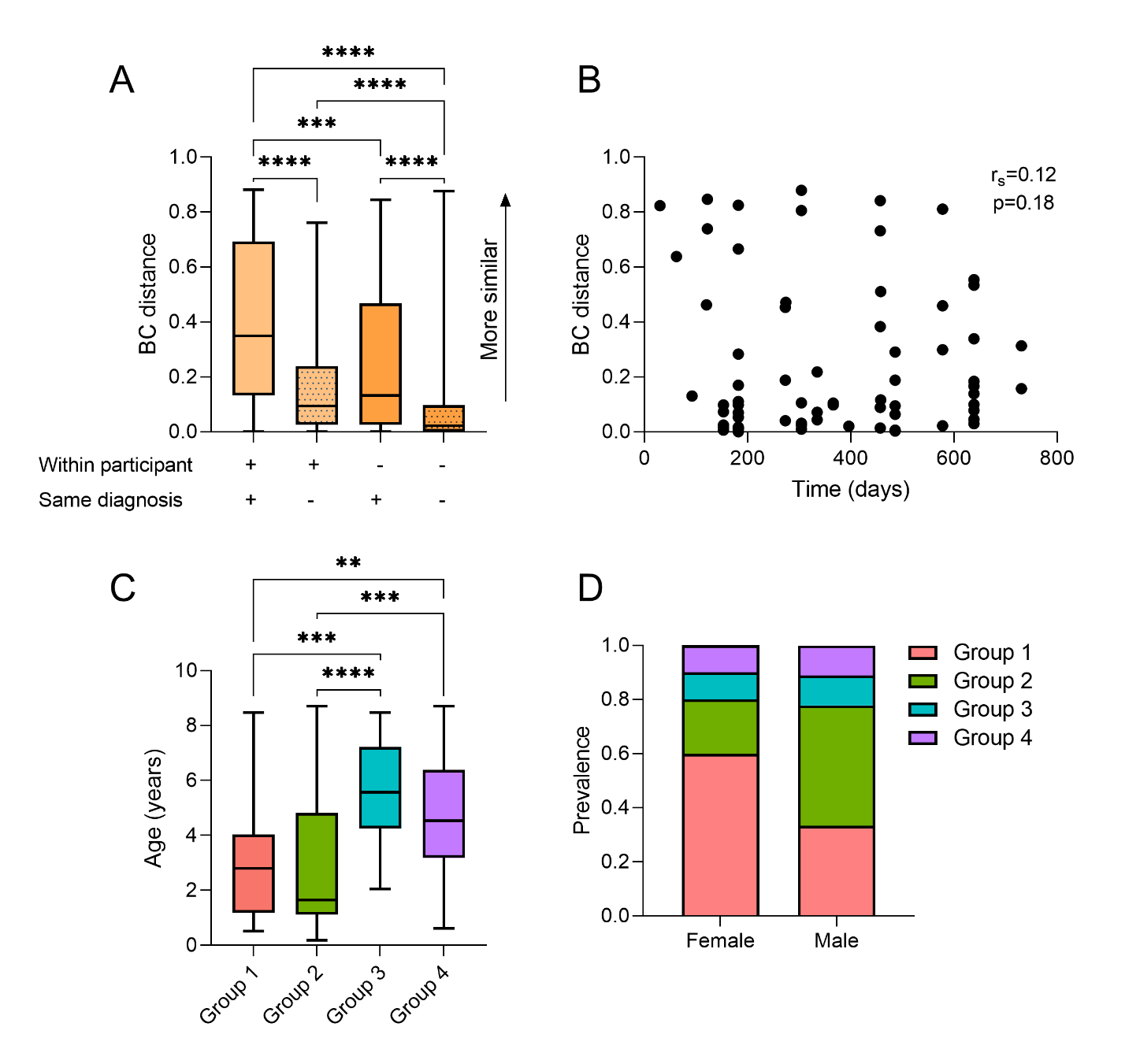


**Supplementary Figure 3:** A) Similarity of ear microbiota compositions (BC distance) where greater distance = samples are more similar. Within participant (+) = similarity of a participant’s left ear compared to right ear; Within participant (-) = participant’s left or right ear compared to left or right ear of all other participants; Same diagnosis (+) = both ears diagnosed with either acute otitis media (AOM), otitis media with effusion (OME), Acute otitis media with perforation (AOMwP), chronic suppurative otitis media (CSOM), granular myringitis (GM), no ear disease (Normal), eustachian tube dysfunction (ETD), or dry perforation (Dry perf); Same diagnosis (-) = ears with different diagnoses. B) Spearman’s rho correlation between days between sample collection and BC distance. C) Participant age of diagnosis by ear disease groups. D) Maximum disease diagnosed stratified by sex. Group 1: Ear disease with tympanic membrane perforation, Group 2: Ear disease with intact tympanic membranes, Group 3: No ear disease with tympanic membrane perforation, Group 4: No disease and intact tympanic membrane. Between group differences assessed by Kruskal–Wallis test, with Dunn’s post-hoc analysis. **p<0.01, ***p<0.001, ****p<0.0001.

**
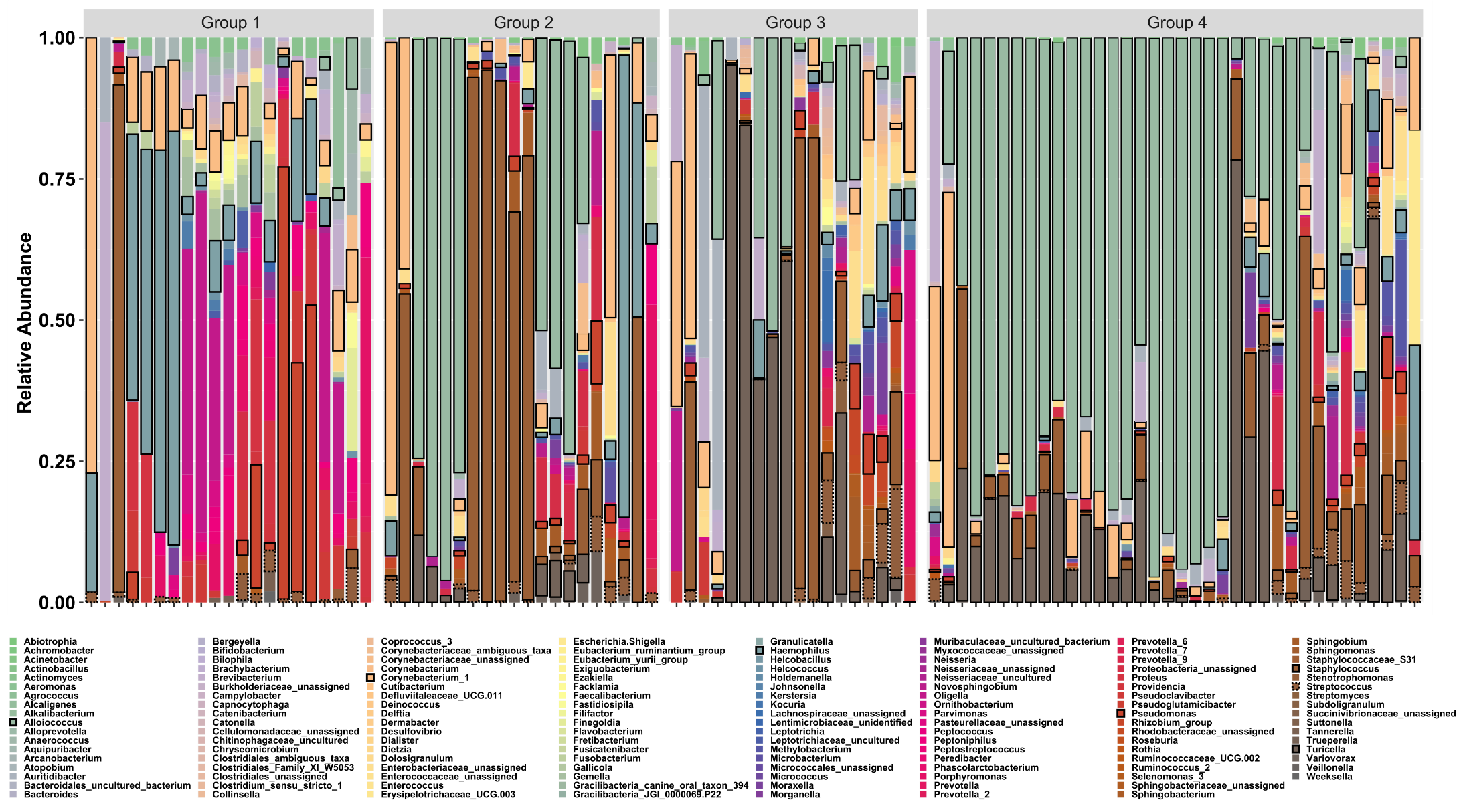
**

**Supplementary Figure 4:** Ear taxa bar plot separated by ear disease groups. Group 1: Ear disease with tympanic membrane perforation, Group 2: Ear disease with intact tympanic membranes, Group 3: No ear disease with tympanic membrane perforation, Group 4: No disease and intact tympanic membrane. Taxa highlighted by bold are those present in >50% of ear swabs.

**
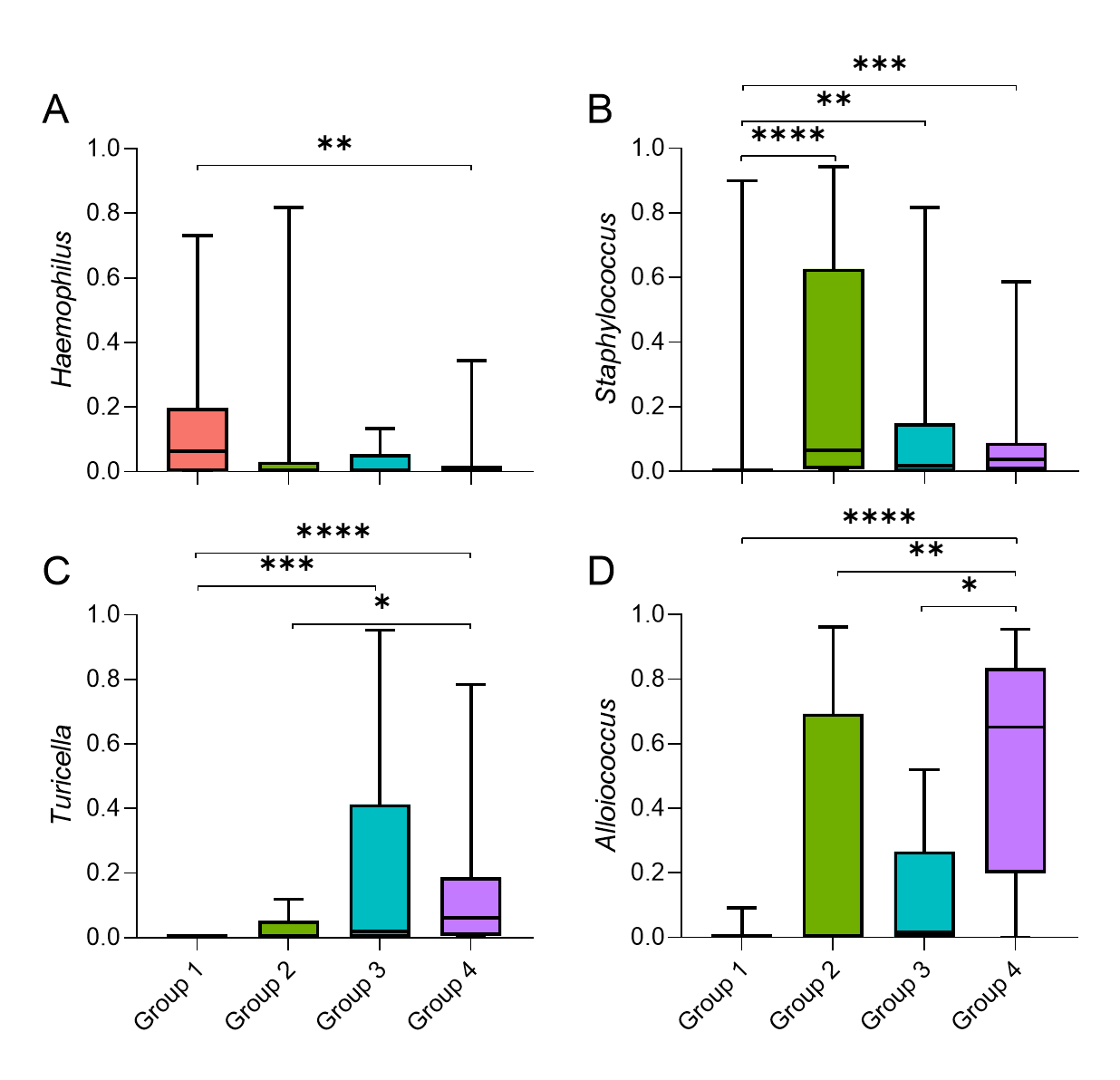
**

**Supplementary Figure 5:** Relative abundance of select genera separated by ear disease groups. Group 1: Ear disease with tympanic membrane perforation, Group 2: Ear disease with intact tympanic membranes, Group 3: No ear disease with tympanic membrane perforation, Group 4: No disease and intact tympanic membrane. Between group differences assessed by Kruskal–Wallis test, with Dunn’s post-hoc analysis. *p<0.05, **p<0.01, ***p<0.001, ****p<0.0001.

**
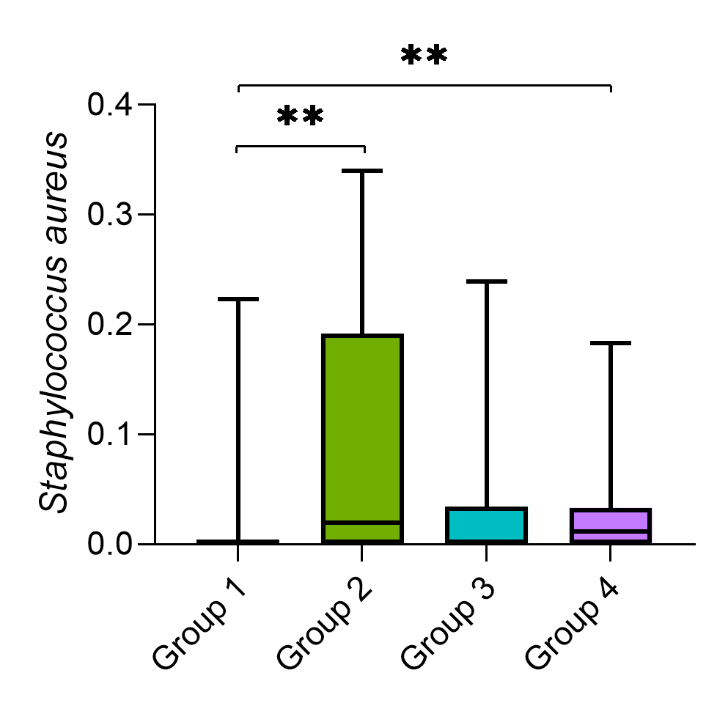
**

**Supplementary Figure 6:** Relative abundance of *Staphylococcus aureus* separated by ear disease groups. Group 1: Ear disease with tympanic membrane perforation, Group 2: Ear disease with intact tympanic membranes, Group 3: No ear disease with tympanic membrane perforation, Group 4: No disease and intact tympanic membrane. Between group differences assessed by Kruskal–Wallis test, with Dunn’s post-hoc analysis. **p<0.01.
